# Comparative Genomic Hybridization (CGH) Reveals a Neo-X Chromosome and Biased Gene Movement in Stalk-Eyed Flies (Genus *Teleopsis*)

**DOI:** 10.1371/journal.pgen.1001121

**Published:** 2010-09-16

**Authors:** Richard H. Baker, Gerald S. Wilkinson

**Affiliations:** 1 Sackler Institute for Comparative Genomics, American Museum of Natural History, New York, New York, United States of America; 2 Department of Biology, University of Maryland, College Park, Maryland, United States of America; National Institute of Genetics, Japan

## Abstract

Chromosomal location has a significant effect on the evolutionary dynamics of genes involved in sexual dimorphism, impacting both the pattern of sex-specific gene expression and the rate of duplication and protein evolution for these genes. For nearly all non-model organisms, however, knowledge of chromosomal gene content is minimal and difficult to obtain on a genomic scale. In this study, we utilized Comparative Genomic Hybridization (CGH), using probes designed from EST sequence, to identify genes located on the X chromosome of four species in the stalk-eyed fly genus *Teleopsis*. Analysis of log_2_ ratio values of female-to-male hybridization intensities from the CGH microarrays for over 3,400 genes reveals a strongly bimodal distribution that clearly differentiates autosomal from X-linked genes for all four species. Genotyping of 33 and linkage mapping of 28 of these genes in *Teleopsis dalmanni* indicate the CGH results correctly identified chromosomal location in all cases. Syntenic comparison with *Drosophila* indicates that 90% of the X-linked genes in *Teleopsis* are homologous to genes located on chromosome 2L in *Drosophila melanogaster*, suggesting the formation of a nearly complete neo-X chromosome from Muller element B in the dipteran lineage leading to *Teleopsis*. Analysis of gene movement both relative to *Drosophila* and within *Teleopsis* indicates that gene movement is significantly associated with 1) rates of protein evolution, 2) the pattern of gene duplication, and 3) the evolution of eyespan sexual dimorphism. Overall, this study reveals that diopsids are a critical group for understanding the evolution of sex chromosomes within Diptera. In addition, we demonstrate that CGH is a useful technique for identifying chromosomal sex-linkage and should be applicable to other organisms with EST or partial genomic information.

## Author Summary

The distribution and organization of genes on chromosomes vary widely among animals. Chromosomes can change in number and size, as well as gene composition, over short evolutionary time scales. Furthermore, chromosome location can influence how genes are expressed in various tissues and how they evolve. The sex chromosomes, in particular, have a dynamic impact on gene movement, expression, and evolution. Uncovering the chromosomal location of genes has traditionally been difficult for non-model organism species. In this study, we assess sex chromosome linkage using a new method that hybridizes DNA from males and females to probes representing over 3,400 genes in stalk-eyed flies. This technique identifies 533 genes (15%) that are located on the X chromosome with the remaining genes located on two autosomes. Comparison of these genes with their location in *Drosophila* indicates that the X chromosome in stalk-eyed flies is nearly completely homologous to the autosome 2L in *D. melanogaster*. This result reveals the formation of a neo-X chromosome in the lineage leading to stalk-eyed flies and indicates that stalk-eyed flies provide a valuable new system to study the origin and evolution of sex chromosomes.

## Introduction

The origin and evolution of sex chromosomes have long been of interest to geneticists and evolutionary biologists [1]–[3]. Besides their essential role in sex-determination, sex chromosomes are distinct from autosomes in their gene content, pattern of gene expression, rate of gene duplication and rate of protein evolution [4]. These differences are critical in shaping both the evolution of sexual dimorphism and patterns of speciation. Dramatic variation exists among animals in sex determination systems [3] and genomic organization of sex chromosomes [5]. Despite this variation, sex chromosome gene content is unknown for most species and only in a handful of taxonomic groups (e.g. mammals, *Drosophila*) are there sufficient comparative data to map the dynamics of sex chromosome evolution [6]–[12]. In this paper, we explore the utility of Comparative Genomic Hybridization (CGH) microarray experiments to differentiate X-linked genes from autosomal genes in several species of stalk-eyed flies from the genus *Teleopsis*. This analysis reveals the formation of a neo-X chromosome in *Teleopsis* relative to other Diptera, and identifies substantial gene movement between autosomes and sex chromosomes.

Considerable theory [e.g. 13]–[17] has been developed under the assumption that sex chromosome identity is determined by the presence of a male-determining factor that initially appeared on an autosome. Once genic sex determination appears, sexually antagonistic alleles, i.e. those with beneficial effects in males but harmful effects in females, should increase in frequency if they are linked to the sex-determining factor [15]. As soon as multiple loci are required for male functionality (or if there are alleles that cause sterility in the opposite sex), then selection will favor reduced recombination between the sex chromosomes. Lack of recombination, together with the joint effects of mutation, natural selection and genetic drift, are then expected to result in degeneration of the Y chromosome (or W in female heterogametic species). Eventually, according to theory, extreme divergence in gene content between sex chromosomes will evolve with only a small number of genes essential for male function expected to survive on the Y.

Once sex chromosomes appear, dosage compensation mechanisms will often evolve to equalize the gene expression differences that result from one sex being heterogametic [18], [19]. In addition, selection for how genes are expressed on sex chromosomes relative to autosomes is expected to differ because X chromosomes are exposed to selection in males half as often as in females, assuming an equal sex ratio. Conflict will arise when alleles that favour one sex are harmful to the other. How such sexually antagonistic conflict is resolved depends on the dominance of the alleles [15], [20]. A number of recent studies have attempted to evaluate these predictions and so far, largely support predictions based on dominant, rather than recessive, allelic effects. In *Drosophila*, a large fraction of the genome exhibits sex-biased expression [21], [22] and X chromosomes show evidence of feminization, i.e. there are fewer male-biased genes and more female-biased genes on the X chromosome than on the autosomes and much of the bias in gene expression is associated with gametogenesis [23]–[25].

An alternative, but not mutually exclusive, explanation for why genes with male-biased expression are under-represented on the X chromosome is that this chromosome is transcriptionally silenced during spermatogenesis in flies [26]–[28] and mammals [29]. Meiotic sex chromosome inactivation (MSCI) has been used to explain why gene movements caused by retrotransposition (detectable by the absence of introns in the derived copy) more commonly involve movement from X chromosomes to autosomes than the reverse and typically exhibit expression in *D. melanogaster* testes [30]. Similar patterns have been found for several other *Drosophila* species in which genes have moved by both RNA and DNA-based mechanisms [9], [31]. Movement of mammalian genes from the X to autosomes has also been linked to sex chromosome silencing [10], [32]. Furthermore, the demasculinization of a neo-X chromosome in *D. pseudoobscura* appears to be driven not by shifts in sex-biased gene expression but rather by differential gene gain, loss and movement [25]. Male-biased genes on the neo-X of *D. pseudoobscura* have not evolved female-biased expression. Instead, they have been either lost from the X and then newly created on an autosome or moved from the X to an autosome.

Stalk-eyed flies in the family Diopsidae are an excellent group in which to explore the dynamics of sex chromosome evolution. They have become an iconic system for studying sexual selection and the sex chromosomes harbor much of the genetic variation affecting numerous aspects of their reproductive biology. Many species exhibit dramatic sexual dimorphism in head shape in which the eyes are placed on the ends of stalks [33]. In some males, the outer most distance between the eyes (eyespan) is twice their body length. Sexual dimorphism in eyespan has evolved multiple times within the family [34]. Evidence suggests that long eyespan males in sexually dimorphic species succeed in male-male contests [35] and are also preferred by females [36]–[38]. Much of the genetic variation associated with exaggerated male eyespan in at least one dimorphic species, *Teleopsis dalmanni*, is located on the X chromosome [39], [40]. In addition, X chromosome effects have also been found for sperm length [41] and parts of the female sperm storage organs [42].

An additional noteworthy feature of diopsids from southeast Asia is that several species in the genus *Teleopsis* [recently synonymized with *Cyrtodiopsis*, 43] exhibit a classic sex ratio polymorphism in which male carriers produce predominantly female offspring [44], [45]. This meiotic drive system is genetically linked to eyespan such that male eyespan serves as an indicator of genetic quality due to an association between short eyespan and segregation distortion [46]. In *T. dalmanni*, the loci influencing drive is X-linked and appears to be associated with an inversion complex that restricts recombination on this chromosome [40]. Therefore, knowing which genes reside on the X chromosome in *T. dalmanni* would be extremely valuable for understanding the genetic consequences of sexual selection in the diopsids and provide another invertebrate system to compare with *Drosophila*. CGH allows the identification of sex-linkage on a substantially larger scale than has been possible to date by traditional linkage mapping.

CGH microarray analysis is primarily used for fine scale identification of gene copy-number variation and is most commonly used in medical applications [47]–[49]. Because the technique involves the relatively simple approach of hybridizing DNA from two different tissues that may differ in gene copy-number at one or several regions of the genome, we expected that it might accurately distinguish the copy-number difference in X-linked genes between male and female DNA samples. Female stalk-eyed flies are XX and, therefore, should produce a hybridization intensity for X-linked genes that is twice that of males, which are XY. Alternatively, autosomal genes should exhibit no difference between the sexes, while Y-linked genes will produce a hybridization signal only for males. Therefore, oligonucleotide arrays were generated from an annotated EST library for *T. dalmanni* and genomic hybridization comparing male and female DNA were performed for this species and three congeneric species.

## Results

### *T. dalmanni* Chromosome Location

**Figure 1:**
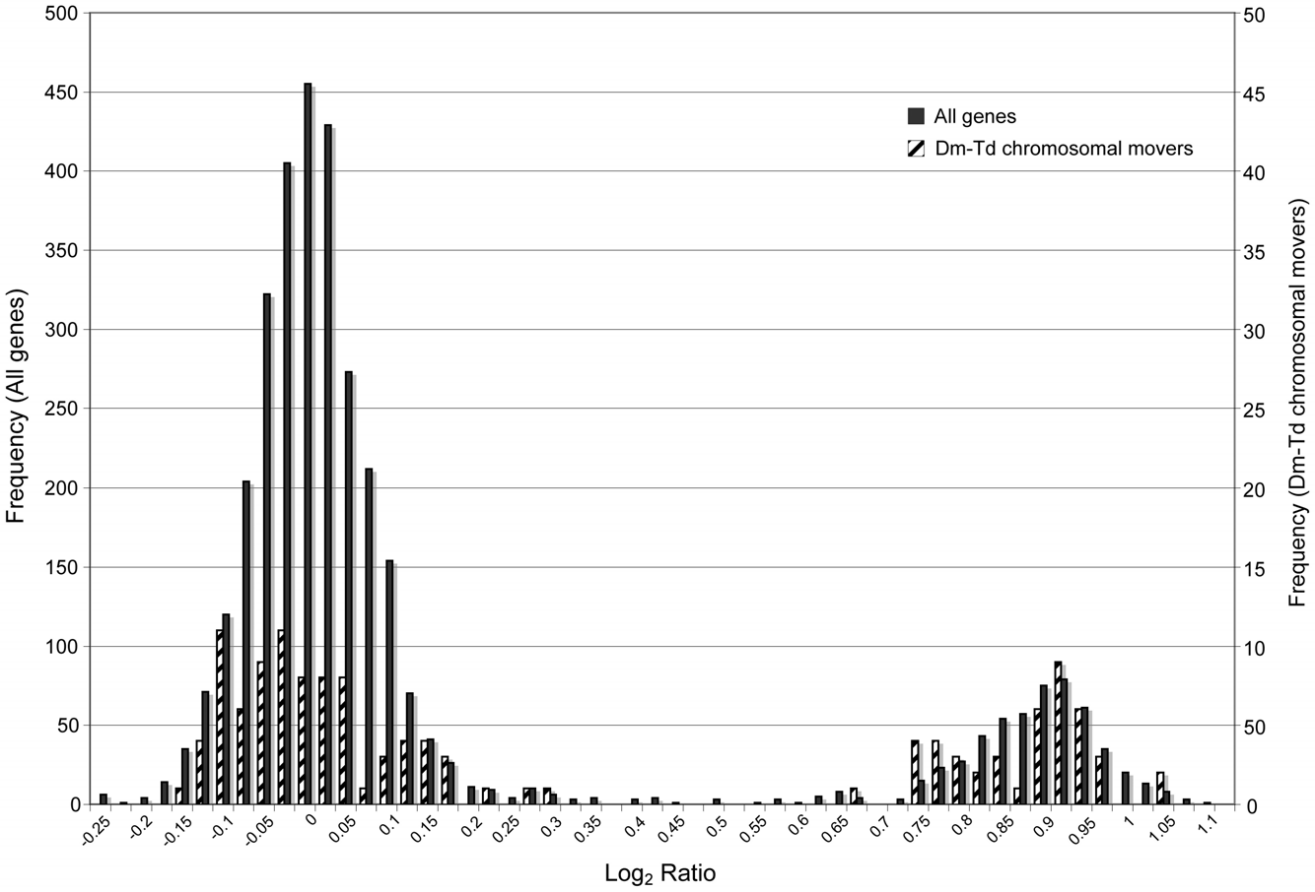
Distribution of *T. dalmanni* CGH log_2_ ratio values. Male and female genomic DNA were hybridized to microarray slides containing probes designed from EST sequence data. A total of 3444 genes are represented. The large peak indicates autosomal genes while the smaller peak are genes on the X chromosome. Dark bars indicate the log_2_ ratio distribution for all the genes, while the hatched bars provide the log_2_ ratio distribution for the 132 genes that violate the syntenic relationship between the X chromosome in *T. dalmanni* and the 2L chromosome in *D. melanogaster*.

**Figure 2:**
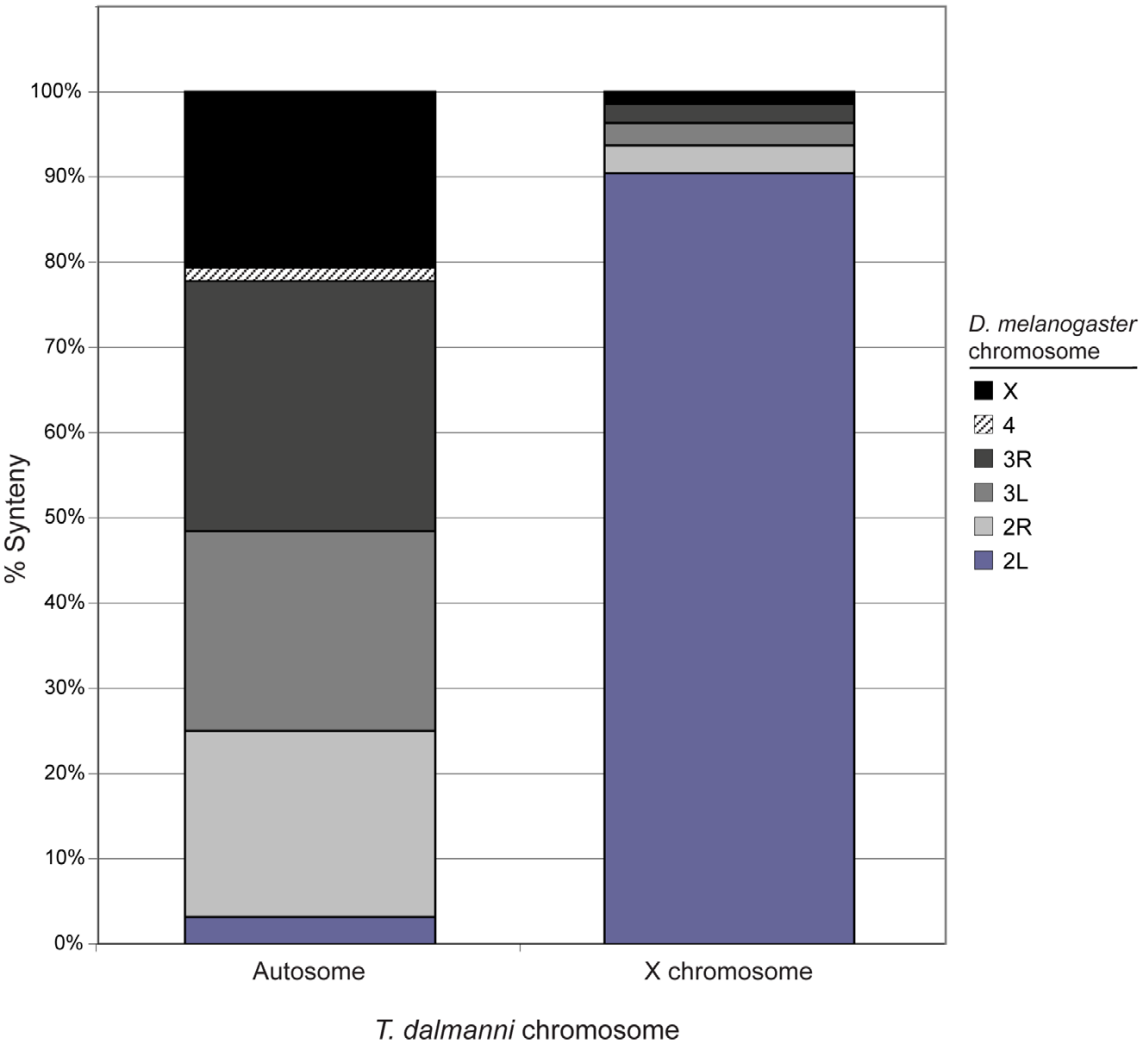
*T. dalmanni* chromosomal synteny with *D. melanogaster*. The autosomal category includes 2891 *T. dalmanni* genes and the X chromosome comprises 533 *T. dalmanni* genes. Homology between *D. melanogaster* and *T. dalmanni* was assessed based on BlastX searches of EST sequence [50].

The *T. dalmanni* log_2_ ratio values of female-to-male signal intensities for 3444 genes, averaged across four hybridizations, exhibit a clear bimodal distribution with one peak at 0 and the second peak at 0.925 (Figure 1; Table S1). The average correlation in gene log**_2_** ratio values across different hybridizations was 0.938. Based on the histogram, the interval with the fewest entries (5 genes) fell between 0.45 and 0.55 so a cut-off of 0.5 was used to distinguish autosomal from X-linked genes. With this criterion, 2891 genes were scored as autosomal, 533 as X-linked and 16 as unknown. Therefore, based on this sample of genes, the X chromosome represents approximately 15% of the *T. dalmanni* genome, a measure that is similar to an estimate of the relative size of the X chromosome (12%) generated from mitotic chromosome lengths [Figure 2, 39]. K-means clustering did not provide any chromosomal assignments that contradicted the first method but scored most of the unknown genes from the first method as X-linked. Overall, K-means scored 2895 genes as autosomal and 545 genes as X-linked. We validated chromosome classifications for 33 loci, four of which were putatively on the X and one was on the Y chromosome. After PCR and genotyping of at least 35 males and 35 females, all loci were assigned correctly with 28 putative autosomal loci containing male heterozygotes, and 4 putative X-linked loci lacking male heterozygotes (Table S2). One gene, ORF-126, had an extremely low log**_2_** ratio of −6.732 and the next lowest value for any gene was −0.569. Therefore, we suspected this gene was Y-linked and designed primers from the EST sequence to test this possibility. We conducted PCRs each on 47 individual males and 47 individual females and found that all of the male PCRs produced a band while none of the female PCRs produced a band, confirming the location of ORF-126 on the Y chromosome.

### Synteny with *Drosophila*

Inspection of the gene annotation for the *D. melanogaster* genes that are homologous to the *T. dalmanni* genes on the array reveals that 453 (90%) of the X-linked genes in *T. dalmanni* are found on chromosome 2L in *D. melanogaster* (Figure 2). The remaining 46 X-linked genes that have a homologous gene in *D. melanogaster* are found on each of the other major chromosome arms in roughly equal numbers (15 from 2R, 11 from 3L, 11 from 3R and 9 from the X). Conversely, the *T. dalmanni* autosomal genes have relatively few homologs on 2L (86 genes) compared to the other major *Drosophila* arms: 2R (601), 3L (646), 3R (805), and X (569) (Figure 2). This pattern indicates a strong syntenic relationship between the X chromosome in *T. dalmanni* and chromosome 2L in *D. melanogaster*. Figure 1 depicts the log ratio values for the genes that violate this syntenic relationship. If the putative chromosomal movement indicated for these genes is an artifact of the methodology we would expect their log**_2_** ratios to be concentrated more in the valley between the peaks but this is not the case. Using GeneMerge, for the small number of genes whose homologs are not located on 2L in *D. melanogaster* but are X-linked in *T. dalmanni* we tested whether their location on the non-2L chromosome in *D. melanogaster* is clustered within a specific chromosomal region on that chromosome. The program divides each chromosome into regions corresponding to the cytogenetic map designations and assesses whether a subset of genes in a sample are disproportionately represented in one of these regions. A clustered distribution might suggest the existence of a small primitive X chromosome that was subsequently fused with the 2L homolog, but the non-2L X-linked genes were randomly distributed across each of their respective homologous chromosome in *D. melanogaster*.

**Figure 3:**
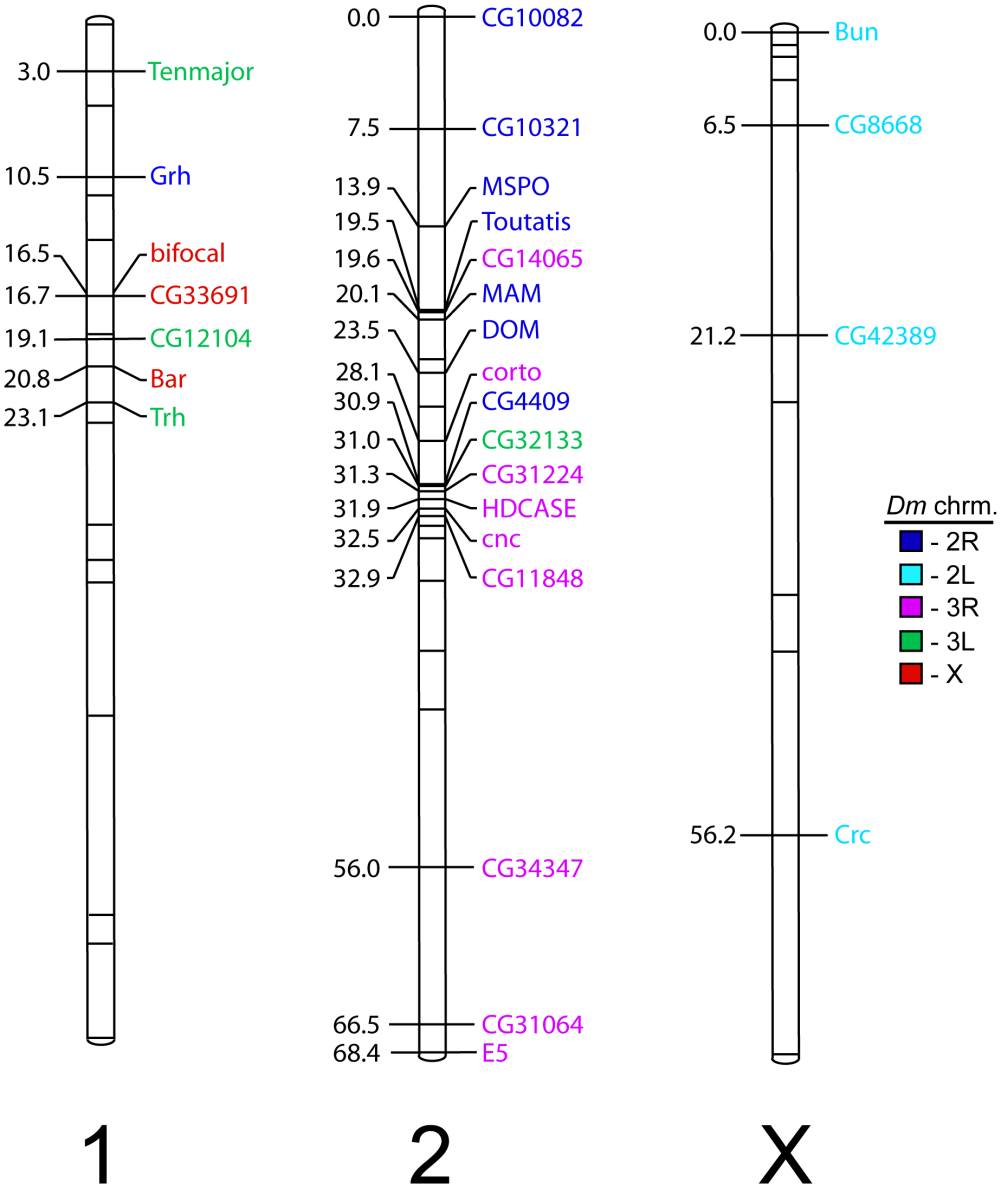
Chromosomal linkage map of 28 annotated EST genes in *T. dalmanni*. Unlabeled hashmarks indicate positions of microsatellite markers. This map was created using genotypes of 639 flies (288 males, 351 females) from two F2 families obtained by crossing lines of flies selected for increased or decreased eye stalk length [cf. 40]. 23 markers were informative in both families and provided the framework for estimating linkage relationships for markers that were only informative in a single family. Color-coding indicates the chromosomal arm location in *D. melanogaster*. As indicated by CGH, four X-linked loci are found on 2L. Furthermore, one autosome appears to be a fusion of X+3L while the other autosome contains genes predominantly from 2R and 3R.

In order to investigate the synteny between the non-2L chromosomes in *D. melanogaster* and the two autosomes in *T. dalmanni* we mapped 28 genes by genotyping flies from two previous F2 intercross experiments involving lines in which males were selected for increased or decreased relative eyestalk length [40]. Each of the genes contained a single amino acid repeat region that varied in length between the lines and, therefore, segregated as a length polymorphism. Inspection of the resulting linkage map (Figure 3, Table S2) reveals that one diopsid autosome contains multiple genes from the *D. melanogaster* X and 3L chromosome arms (Muller elements A and D) while the other autosome contains multiple genes from chromosome arms 2R and 3R (Muller elements C and E). Two genes (*grainy head* and *CG32133*) appear to have moved between *Teleopsis* autosomes based on their location in *D. melanogaster*.

### Gene Movement in *Teleopsis*

**Figure 4:**
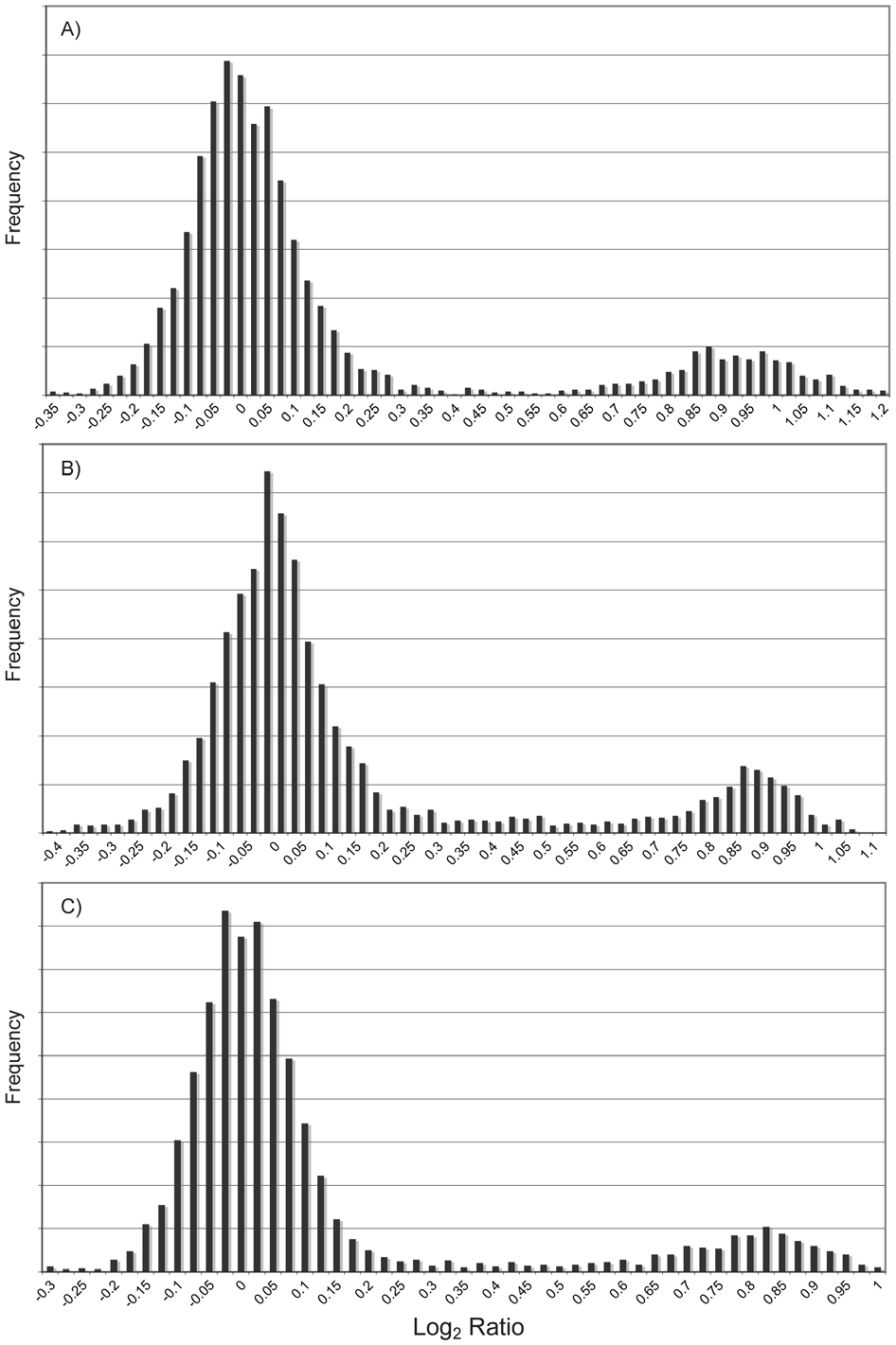
Distribution of CGH log_2_ ratio values for three other *Teleopsis* species. Male and female genomic DNA were hybridized to microarray slides containing probes designed from *T. dalmanni* EST sequence data. Approximately 3400 genes are represented in each histogram. The large peak indicates autosomal genes while the smaller peak are genes on the X chromosome. A) *Teleopsis whitei*. B) *Teleopsis thaii*. C) *Teleopsis quinqueguttata*.

In addition to the *T. dalmanni* slides, we performed CGH experiments on three congeneric species—*T. whitei*, *T. thaii* and *T. quinqueguttata*. In all cases, cDNA from these species was hybridized to the microarray slide that contained probes generated from the *T. dalmanni* EST sequences. All three species exhibited a similar bimodal distribution of log**_2_** ratio values (Figure 4; Table S1) but with slightly reduced separation between the peaks. As a result, more genes were scored as ‘unknown’ for each of these species than for *T. dalmanni*. *T. whitei*, the species that is most closely related to *T. dalmanni*, had the fewest number of unknown genes (56) followed by *T. quinqueguttata* (144) and *T. thaii* (225). The average correlation in log**_2_** ratio values across different hybridizations (0.917 for *T. whitei*, 0.737 for *T. thaii* and 0.811 for *T. quinqueguttata*) was also lower for these species than in *T. dalmanni*. The vast majority of genes (93.7%) that are autosomal in *T. dalmanni* are also autosomal for the other *Teleopsis* species, indicating strong syntenic conservation within the genus. As with the *T. dalmanni* analysis, the K-means clustering of the data for the other *Teleopsis* species produced chromosome designations similar to the method based on the confidence intervals of individual probes. The K-means analysis scored the majority of the unknown genes as X-linked and in a few cases (5 genes in *T. whitei*, 4 genes in *T. thaii*, and 6 genes in *T. quinqueguttata*) scored a gene that was autosomal in the first analysis as X-linked. As with ORF-126 in *T. dalmanni*, there were two genes, *RNA polymerase II 215kD subunit* (*RpII215*) and a paralogous copy of *Cuticular protein 35B* (*Cpr35B*) which has duplicated several times within diopsids [50], in *T. quinqueguttata* that had extremely low log**_2_** ratio values, −5.107 and −4.378 respectively. PCR of 48 males and 48 females in *T. quinqueguttata* confirms a Y chromosome location for *RpII215* but we have been unable to verify the location of the *Cpr35B* paralog through PCR presumably because we have not yet been able to design copy-specific PCR primers.

**Figure 5:**
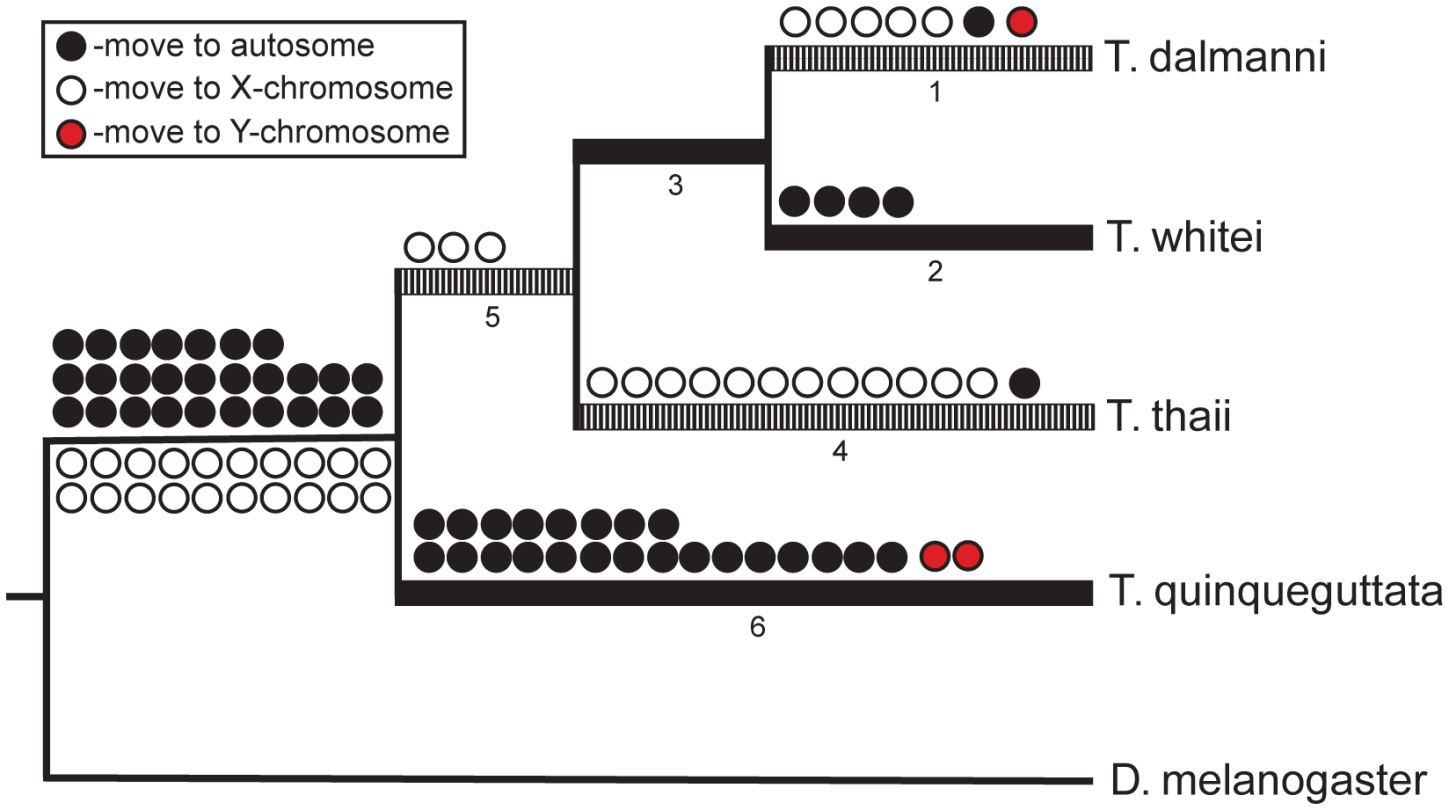
Gene movement in *Teleopsis*. Reconstruction of gene movement between autosomes and the X chromosome was polarized using chromosomal data from *A. gambiae* and *D. melanogaster*. The X chromosome in *Teleopsis* was assumed to be homologous to chromosome 3R in *A. gambiae* and 2L in *D. melanogaster*. The branches with hash marks represent those that have undergone increases in eye-stalk sexual dimorphism. The states for each species and the specific genes associated with each reconstruction (using the branch numbers as reference) are presented in Table S2.

Assuming a syntenic relationship between *D. melanogaster* 2L and the *Teleopsis* X chromosome (and between all other *D. melanogaster* chromosomes and the *Teleopsis* autosomes) and using the *D. melanogaster* and *Anopheles gambiae* chromosomal designations to polarize the reconstruction within *Teleopsis*, we reconstructed the pattern of gene movement on and off the X chromosome (Figure 5; Table S3). A total of 193 genes exhibit movement either between *Drosophila* and *Teleopsis* or within *Teleopsis*. Twenty-nine genes were not assigned a specific pattern of movement either due to a lack of homology with *D. melanogaster* and *A. gambiae* or because they produced ambiguous reconstruction within *Teleopsis*. For 65 of the genes that violated the syntenic relationship between *D. melanogaster* 2L and the *Teleopsis* X, it was more parsimonious, based on the syntenic relationship with *A. gambiae*, to ascribe the gene movement as occurring within the lineage leading to *Drosophila* rather than to *Teleopsis*. Of the remaining genes, there are a similar number of genes that have moved on to an autosome (27) as on to the X chromosome (20) in the lineage leading to *Teleopsis*. Within *Teleopsis*, movement of genes from the X chromosome to an autosome is concentrated (23 out of 29 gene moves) on the branch leading to the monomorphic taxa, *T. quinqueguttata*, while all of the movement of genes off of the autosomes and onto the X chromosome occurs on the branches associated with the two most sexually dimorphic taxa, *T. thaii* and *T. dalmanni* (Figure 5). Analysis of the gene annotation associated with the pattern of gene movement indicates that the set of genes that have moved onto the X chromosome are overrepresented (P = 0.049) for genes that function in the ‘structural constituent of ribosome’ (GO:0003735). This pattern is generated primarily by *T. thaii* as 5 of the 12 genes that have moved onto the X chromosome on the branch leading to this species are involved in this molecular function. Genes that had moved on to an autosome were not overrepresented for any functional category.

**Figure 6:**
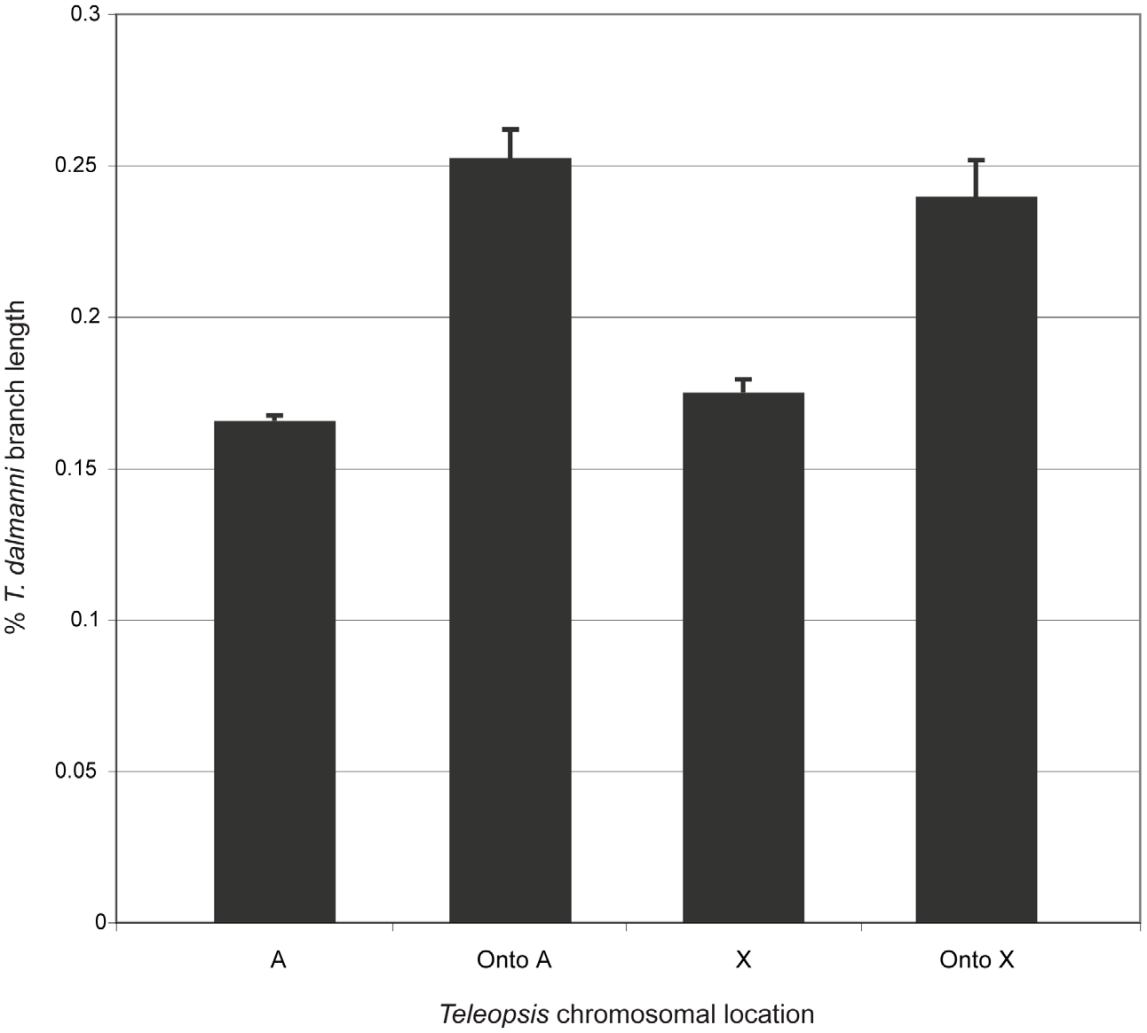
Relationship between *Teleopsis* chromosomal location and the rate of protein evolution. The rate of protein evolution in *Teleopsis* was measured as the branch length leading to *T. dalmanni* expressed as a percentage of the entire tree length based a phylogenetic analysis including *A. gambiae* and three *Drosophila* species [50]. The sample sizes for the chromosomal locations are: A - 2034 genes, Onto A – 41 genes, X – 342 genes and Onto X – 23 genes.

Several studies in *Drosophila* have demonstrated that chromosomal location and the pattern of gene movement across chromosomes are associated with differential rates of protein evolution [51], [52], duplication [9], [31] and divergent patterns of gene expression [22], [24], [25]. Estimates of each process were obtained for *Teleopsis* in an analysis of the eye-antennal imaginal disc transcriptome [50]. Therefore, we examined the relationship between these variables and gene location within the genus. The rate of protein evolution was measured as the percentage of amino acid change in the lineage leading to *T. dalmanni* relative to the amount of change for that gene in the *Drosophila* and *Anopheles* lineages. Using this index, there is little difference between genes that reside exclusively on the autosomes or the X chromosome in *Teleopsis*, but genes that have moved between these chromosomes are evolving significantly faster than those that have not moved (P<0.0001; Kruskal-Wallis test; Figure 6). This pattern is also supported if we look just at the genes that were determined, by a relative rate test, to be evolving significantly faster at the protein level in *T. dalmanni* than in *D. melanogaster*, *D. pseudoobscura* and *D. virilis* [50]. These genes are overrepresented in the set of genes that have moved chromosomes, particularly those that have moved onto the autosomes (χ^2^ = 27.74, P<0.0001; Chi-squared test). 2.62% of all genes were classified as evolving significantly faster in *T. dalmanni*, but 19.51% of the genes that have moved onto an autosome fell into this category, compared with 8.33% for the genes that have moved onto the X chromosome, 4.11% for exclusively X-linked genes and 1.97% for genes that are exclusively autosomal.

The analysis of the eye-antennal imaginal disc EST database revealed 20 putative duplication events in the *Teleopsis* lineage [50]. An additional duplication that was not detected in that study, involving the sex determination gene *transformer 2* (*tra2*), was revealed by the CGH results. Hybridization to probes generated from different consensus sequences (conseqs) that are both homologous to *tra2* revealed that one sequence resides on an autosome while the second sequence is X-linked. Subsequent phylogenetic analysis of the divergence in protein sequence between the conseqs and *Drosophila* homologs confirms the CGH results. Of these 21 putative duplicates, 11 of their homologs in *D. melanogaster* reside on 2L while the remaining homologs are distributed in similar numbers among 2R (2), 3L (2), 3R (5), and X (1). This chromosome distribution is significantly overrepresented for genes on 2L (χ^2^ = 14.143, P = 0.0002; Chi-squared test). Given the syntenic relationship between 2L in *D. melanogaster* and the X chromosome in *Teleopsis*, there is also a significant relationship between gene duplication and residence on the X chromosome in *T. dalmanni* (χ^2^ = 6.430, P = 0.011; Chi-squared test), but the relationship is weaker because three of the duplicates have moved off the X chromosome in *T. dalmanni*. Overall, genes that belong to a duplicate set are significantly more likely to move between chromosomes than are other genes. There are 10 occurrences (27.7%) of genes in the duplicate sets moving chromosomes within *Teleopsis* compared to 88 (2.6%) chromosomal movements for the remaining genes (χ^2^ = 31.031, P<0.0001; Chi-squared test). Of the 10 duplicate gene movements, 7 are off of the X chromosome onto an autosome, two are off an autosome onto the X, and one is onto the Y chromosome. The EST study also compared the gene expression levels in male *T. dalmanni* eye-antennal imaginal discs between lines of flies that had been selected for over fifty generations for increased and decreased eye span and found over 350 differentially expressed genes. These genes, however, were not preferentially associated with chromosomal location (χ^2^ = 3.502, P = 0.174; Chi-squared test) or gene movement (χ^2^ = 2.322, P = 0.508; Chi-squared test).

## Discussion

The results from this study indicate that CGH microarrays are an effective technique for identifying sex chromosome linkage in non-model organisms for which EST or partial genomic information is available. For all four *Teleopsis* species examined, the log**_2_** ratio values of female-to-male hybridization intensities exhibited a distinct bimodal distribution, although there is more ambiguity in the results for the taxa more distantly related to *T. dalmanni*, the species whose EST sequence was used to design the microarray slide. We scored 3444 *Teleopsis* genes as either autosomal or linked to a sex chromosome and confirmed assignment for 33 genes by direct genotyping. The X chromosome comprises approximately 15% of the genome in these species. Comparison with *Drosophila* indicates there is a strong syntenic relationship between the X chromosome in *T. dalmanni* and chromosome 2L in *D. melanogaster*. Both the overall syntenic relationship, as well as the subset of genes that violate this relationship, are supported by the hybridizations in all four species. In addition, the distribution of log**_2_** ratio values of the genes that appear to have moved chromosomes between *Drosophila* and *Teleopsis* is similar to the remaining genes (Figure 1), suggesting this putative chromosomal movement is not an artifact of the methodology. The CGH approach also identified one Y-linked gene in *T. dalmanni* and at least one (and possibly two) Y-linked genes in *T. quinqueguttata*. The evolutionary history and genomic organization of the Y chromosome have received considerable interest recently [53]–[57], but Y-linked genes are difficult to isolate using standard genomic techniques (such as BAC libraries) because of their heterochromatic structure. The CGH approach has considerable promise for identifying genes on the Y chromosome, especially if EST information is available from tissue such as testes, which are likely to be a rich source of Y-linked gene expression [58].

### Evolution of Sex Chromosomes

Diptera provide one of the most comprehensive model systems for studying the differentiation of sex chromosomes. The regulatory network controlling sex determination in *Drosophila* is well characterized at the genetic and molecular level [59]. While some aspects of this system appear unique to this clade, there are other features that are highly conserved across Diptera and provide a valuable comparative framework for examining the genetics controlling sex determination in other fly species [60]–[64]. There is also substantial variation in both sex chromosome composition and sex determination systems at various taxonomic levels within the order [59], [64]–[66]. For instance, Tephritidae include species with isomorphic chromosomes and female heterogamety [67] and, within *Musca domestica*, the chromosomal location of the male-determining factor varies between populations, occurring in some cases on the X chromosome and, in other populations, on one of several autosomes [68], [69]. Despite the substantial variation in these systems, a comprehensive catalog of genes on the sex chromosomes exists for only *Drosophila* and the mosquito, *A. gambiae* [70]. Therefore the data on chromosomal location presented in this study provide valuable information for reconstructing syntenic relationships and mapping the evolution of chromosomal organization within Diptera. Furthermore, the discovery of a neo-X chromosome in *Teleopsis* means that these flies represent a critical group in a comparative analysis of sex chromosome organization and provide a complementary model system for understanding key aspects of sex chromosome evolution.

Because of the abundant interspecific variation in sex chromosome organization and the paucity of information regarding the specific gene composition of these chromosomes within Diptera, it is difficult to reconstruct the steps that may have led to the formation of the neo-X chromosome in *Teleopsis*. There is substantial syntenic conservation between the X chromosomes of *A. gambiae* and *D. melanogaster* [70], [71], flies that share a common ancestor approximately 250 million years ago and represent a more ancestral split than *Teleopsis* and *Drosophila*. However, tephritids, which are thought to be a closer relative of diopsids than are *Drosophila* [72], appear to have a greatly reduced X chromosome containing few genes and a sex determination system controlled by a male-determining factor [67]. This pattern, combined with the fact that the calyptrate flies examined (e.g. *Musca domestica* and *Lucilia cuprina*) have a similar condition, suggests the ancestor leading to diopsids may also have had a small X chromosome and male-determining factor. If this is the case, there are a few scenarios that might describe the formation of the neo-X in diopsids. First, the neo-X may represent the fusion of an autosome to small ancestral sex chromosomes. In this scenario, we would predict that some homology would be maintained between the ancestral portion of the sex chromosome of diopsids (i.e. the region not homologous to 2L) and chromosomal regions in other flies. There was no syntenic relationship between the non-2L X linked genes in *Teleopsis* and any chromosomal region in *Drosophila*, but there may be synteny between these genes and the X chromosomes of tephritids or calyptrates. Second, the male determining factor (M) may have moved from a sex chromosome to an autosome (i.e. the homolog of 2L) and the sex chromosome creation process started anew. Similarly, M may have existed in a polymorphic state with respect to chromosomal location (as in *Musca*) and become fixed on an autosome at some point. In either case, we would predict a lack of homology between the sex chromosome of diopsids and tephritids or calyptrates and M should exhibit different micro-syntenic relationships within diopsids than tephritids or calyptrates. Finally, a new gene that is located on 2L may have become the primary sex determination signal supplementing M. The evolution of sex-determination pathways is generally characterized by modification of components higher up in the genetic hierarchy [73], and Shearman [66] has outlined several processes that would result in the acquisition of a new gene in the sex determination pathway. If this scenario applies to stalk-eyed flies, there will be no homology between the primary sex determination gene in diopsids and the M factor of tephritids and calyptrates. Obviously, it is essential for future research to elucidate the sex determination system in *Teleopsis* and to determine the gene content of sex chromosomes in other dipteran systems such as tephritids and calyptrates.

Regardless of the evolutionary transitions in sex chromosome composition leading to *Teleopsis*, the formation of a neo-X chromosome in this lineage provides an opportunity to examine several important components of sex chromosome evolution, such as the degradation of the Y chromosome and the evolution of dosage compensation. In a few *Drosophila* lineages, the presence of neo-X chromosomes has provided invaluable snapshots into these processes [53], [74]–[76]. The neo-X chromosome in diopsids is likely to be more ancient than those in *D. miranda* [76] or *D. americana* [77], but may be similar in age to the neo-X in *D. pseudoobscura*, which has been estimated at 8–10 million years old [78]. Therefore, *Teleopsis* offers an alternative system to *Drosophila* for examining sex chromosome evolution, but the neo-X chromosome in this species is also distinct from those in *Drosophila* in that it appears to represent a wholesale reconstitution of the X rather than a fusion of an autosomal arm to a substantial preexisting X. Only a single Y-linked gene was identified for *T. dalmanni* by the CGH microarrays, so it is premature to speculate on the evolutionary history of the Y chromosome in *Teleopsis*. However, results from this study, along with images of mitotic chromosome lengths [39], clearly indicate the sex chromosomes in *Teleopsis* are heteromorphic in males. Therefore, a dosage compensation mechanism is expected to exist in this species. The microarray experiment conducted in Baker et al. [50] compared gene expression between males from different lines, and provides an opportunity to test for the presence of dosage compensation. If the X chromosome in males is hyper-transcribed there should be no difference in the level of expression between autosomal genes and X-linked genes, and, in fact, we find no difference in the average signal intensity between these groups (P = 0.782; Wilcoxon test). Intriguingly, none of the male specific lethal (MSL) complex genes that control dosage compensation in *Drosophila* [79] were found in the EST survey of *T. dalmanni*. While their absence may result from random sampling of the transcript population, it is possible that diopsids, like *Sciara* [80], utilize a mechanism of dosage compensation that differs from *Drosophila*.

### Gene Movement in *Teleopsis*

Transposition in *Drosophila* and mammals is characterized by an excess of movement off of the X chromosome [8], [9], [30], [31], [81], [82]. The derived autosomal copies generally exhibit testes expression [8], [9], [30], [31], [81], [82], and therefore, selection to avoid meiotic sex chromosome inactivation (MSCI) has been hypothesized as a primary mechanism driving this pattern [8], [9], [30], [31], [81], [82]. It is unknown whether silencing of X-linked genes during late spermatogenesis occurs in stalk-eyed flies. Overall, the reconstruction of gene movement within *Teleopsis* indicates similar amounts of movement on and off of the X chromosome (Figure 4). However, there was a striking relationship in the pattern of movement with respect to sexual dimorphism in eyespan. Movement of genes onto the X chromosome are concentrated on branches leading to the most dimorphic taxa while movement off the X is associated primarily with the monomorphic species, *T. quinqueguttata* (Figure 4). One possible explanation for this pattern is that sexual selection is operating more strongly on testes function within *T. quinqueguttata* than the dimorphic species, such that the genes moving off the X chromosome in this species are undergoing selection for increased testes expression. Regardless of the selection pressures affecting *T. quinqueguttata*, it is noteworthy that the branches leading to the most dimorphic species are characterized primarily by gene movement onto the X chromosome. If these genes play a role in the evolution of eyestalk dimorphism, then they are unlikely to be affected by MSCI. However, sexual antagonism has also been postulated as a factor driving gene movement off of the X chromosome [24], [25] Therefore, we might expect genes affecting male eyespan to be preferentially located on an autosome but gene movement in the dimorphic species appear directed away from not onto the autosomes. As in *Anopheles* [83], gene expression in *Teleopsis* may not be characterized by a demasculinization of the X chromosome. Consistent with this scenario, the largest QTL influencing variation in male eyespan has been mapped to the X chromosome [40].

Much of the ‘off-of-the-X’ gene movement in *Drosophila* is driven by duplication events in which the derived copy, often through retrotransposition, moves off the X and subsequently acquires testes expression [9], [31]. The majority of gene movements reconstructed in *Teleopsis* cannot be traced to duplication events as complete genomic data is not available for this species. However, for the small number of duplicated genes identified in the EST analysis there was a clear relationship between the duplication process and chromosomal organization. Duplicated genes were concentrated on the 2L/X (*D. melanogaster*/*T. dalmanni*) chromosome and over 10 times more likely to move between chromosomes than genes that had not been duplicated. Furthermore, the pattern of gene movement for these duplicated genes is consistent with the ‘out-of-the-X’ hypothesis (7 movements off of the X versus 2 onto the X) but the sample size is too limited to draw strong conclusions.

Genes that have moved chromosomes also exhibit a faster rate of protein evolution within stalk-eyed flies than genes that have not moved. Theoretical models predict that patterns of divergence of X-linked protein coding genes will be distinct from autosomal genes [4], and several empirical studies have identified differences in substitution patterns between X-linked and autosomal genes [51], [84], [85], [but see 86]–[88]. Using a measure of protein evolution that calculates divergence in *Teleopsis* relative to *Drosophila* and *Anopheles* [50], we found no difference in relative divergence between genes on the X chromosome and genes on an autosome. Genes that had moved between chromosomes, however, have higher levels of divergence than those that have not. Despite the extensive research on gene movement in *Drosophila*, to our knowledge, no study has examined, at the genomic level, the association between gene movement and protein evolution within the genus. There is, however, a positive relationship between male-biased gene expression and rates of protein evolution [23], [51]. Therefore, if gene movement in *Teleopsis* is related to the evolution of sexual dimorphism then these genes may be under more intense selection pressures, and evolving faster, than other genes. Ultimately, it will be necessary to measure levels of sex-specific gene expression and quantify rates of evolutionary change before and after translocation in order to fully understand the factors driving this pattern.

One methodological issue that may influence the pattern of gene movement within *Teleopsis* is the effect of sequence divergence on hybridization intensity. Chromosomal location for the three *Teleopsis* species not including *T*. *dalmanni* were inferred from microarray slides with probes designed from *T. dalmanni* ESTs and the effect of sequence divergence on relative intensity for these species is unknown. Some have suggested that assessment of gene expression levels from cross-species hybridization can be problematic [89], although the resolution needed for CGH arrays is less than that for detecting differential gene expression. We attempted to mitigate some of these concerns by limiting probe design to protein coding sequence and using the median hybridization value for up to ten probes from a single EST contig. However, the number of ambiguous chromosomal designations and the between-slide correlations clearly indicate that the error associated with measuring hybridization intensity increases as the species becomes more distantly related to *T*. *dalmanni*. Therefore, we have taken a conservative approach by only assigning chromosomal location to genes that had consistent signal across all four hybridizations for each species. With respect to the pattern of gene movement in the genus, if probe sequence divergence is influencing hybridization intensities, and thus the reconstruction of gene movement, we would not expect the pattern found in this study where, for two divergent species (i.e. *T. thaii* and *T. quinqueguttata*), all the movement is concentrated in one direction for one species and in the other direction for the second species. More likely, gene movement would be randomly distributed among these branches. In addition, for all of the putative gene movement in *Teleopsis*, the mean rank of relative sequence divergence is higher for the genes where the movement occurs on a branch leading to *T. dalmanni* than branches exclusive to the other three species, suggesting that the relationship between movement and sequence divergence is not an artifact created by hybridization effects. In the future, it will be essential for new hybridizations examining chromosomal location in other diopsid species to use probes designed from sequence data from that particular species, as well as to develop resources for direct genotyping in these species.

## Methods

### Study Organism

Diopsids are one of several families within a group of higher flies known as Acalyptrata, a paraphyletic group that also contains the fly families Drosophilidae and Tephritidae. Relationships among acalyptrate fly families have proven difficult to resolve and are actively under investigation, but current evidence indicates that the Tephritoideae is the most likely sister group to the Diopsoideae, and that these superfamilies likely had a common ancestor with *Drosophila* no more than 76 MYA [72]. Four diopsid species in the genus *Teleopsis*, *T. dalmanni*, *T. whitei*, *T. thaii* and *T. quinqueguttata* were used in this study. The first three species are all sexually dimorphic with respect to eyespan while *T. quinqueguttata* is monomorphic. In *T. dalmanni*, eye span exhibits X-linked inheritance [39] and eye span QTL are tightly linked to a *sex-ratio* factor that results in female-biased brood sex ratios [44], presumably as a result of one or more inversions on the X chromosome [40]. As a consequence, males carrying a *sex-ratio* X chromosome have shorter eye span than other males [40]. They also have shorter sperm [41]. Sex chromosome meiotic drive has been detected in multiple populations of *T. dalmanni* and *T. whitei* [44], [45] in southeast Asia, all of which exhibit some degree of postcopulatory reproductive isolation [90]. Phylogenetic relationships among the four *Teleopsis* species are well supported and sexual dimorphism in eyespan has been mapped on the phylogeny [91], [92]. QTL mapping experiments [40], [93] and visualization of mitotic chromosomes in spermatocytes [39] for *T. dalmanni* indicate that this species has three major chromosomes, two autosomal chromosomes and one set of sex chromosomes characterized by an acrocentric X and smaller Y chromosome.

### Microarray Slide Construction

60-mer Agilent oligonucleotide probes were designed based on contig sequences from an EST library made from the eye-antennal imaginal discs of *T. dalmanni* [50]. This study generated over 33,000 ESTs that assembled into over 3400 contigs with significant homology to a gene in *Drosophila*. Probes were designed for nearly all of these genes along with 168 contigs with open reading frames (ORF) greater than 500 basepairs. Because we were also conducting hybridization on *Teleopsis* species other than *T. dalmanni*, probes were limited to protein coding regions in order to minimize the amount of nucleotide divergence between *T. dalmanni* and the other species, which tends to be higher in the UTR regions of the transcripts. A maximum of 10 probes were designed for each gene. One rationale for selecting this number of probes is that the CGH results provided valuable information for a subsequent study comparing sex-biased gene expression between *T. dalmanni* and *T. quinqueguttata*. For the gene expression study, we wanted probes with the lowest amount of sequence divergence between species and the CGH results provided a means for selecting these probes.

### Hybridization and Analysis of Chromosomal Location

The microarray experiment consisted of 4 replicate hybridizations for each species (the data is available from NCBI via accession number GSE20315). Each hybridization sample consisted of 4 or 5 male or female adult flies that were taken from population cages maintained at the University of Maryland, College Park. After removing wings and heads DNA was extracted from macerated fly bodies with Qiagen DNeasy kits using the insect sample protocol. DNA concentration and quality was then estimated with a Nanodrop ND-1000 spectrophotometer and 3 µg of DNA was used in each sample. Each DNA sample was fractionated by restriction digestion with AluI and RsaI for 2 h at 37°C. Each sample was then labeled with either Cy-3 or Cy-5 and processed according to the Agilent array-based CGH protocol. After hybridization for 24 h at 65°C, arrays were scanned using an Agilent G2539A microarray scanner. Hybridization intensity was measured from array images scanned at each dye wavelength using Agilent's Feature Extraction Software. Features were excluded from further analysis if the majority of pixels were saturated or the median pixel intensity was less than two times the background. Intensity scores were normalized using the linear normalization methodology in the Feature Extraction Software. For each hybridization, we calculated the median log**_2_** ratio (all log**_2_** ratios were defined as female/male intensity) of all the probes for a given gene or ORF. Then, the log**_2_** ratios for a given hybridization were centralized by adding or subtracting a constant value to the median log**_2_** ratios so that the peak of the lower distribution was centered over zero.

Standard CGH software and analysis techniques are not appropriate for this study because a chromosomal map of the genome does not exist for *T. dalmanni*. Therefore, after signal extraction and centralization, we conducted two analyses to separate the gene log**_2_** ratios into two categories (i.e. autosomal or X-linked). In the first method, we generated a histogram of the gene log**_2_** ratios averaged across the four hybridizations for each species. Based on this histogram, intervals of size 0.1 were searched every 0.0125 across the log_2_ distribution to determine the interval with the fewest entries. The value in the center of this interval was then used as a cut-off for distinguishing chromosomal categories. Using the variation in log**_2_** ratios across the four hybridizations, we then calculated a 95% confidence interval for each gene. If the confidence interval of a given gene did not contain the cut-off value, then that gene was assigned as autosomal if its average log**_2_** ratio value was less than the cut-off and X-linked if its average log**_2_** ratio value was greater than the cut-off. If a gene's confidence interval contained the cut-off value then the gene's location was designated as unknown. The second method we used to separate the probes into groups was K-means clustering [94]. This method performs well when specifying an exact number of clusters and, using the program tmev [95] we conducted 50 iterations of average linkage clustering based on the Euclidian distance to classify the genes into 2 clusters. Overall, the K-means analysis produced chromosomal designations that required about 3 times as much gene movement between the autosomes and the X chromosome within *Teleopsis* as the first method. Therefore in order to assess the pattern of gene movement relative to *Drosophila* and within the genus we used the more conservative designations provided by the confidence interval method.

### Analysis of Gene Movement

Reconstruction of gene movement within *Teleopsis* was conducted in MacClade (V4.06) using a simple parsimony approach. Chromosomal locations in *D. melanogaster* were used to root the reconstruction of gene movement within *Teleopsis* assuming a syntenic relationship between chromosome 2L in *D. melanogaster* and the X chromosome in *Teleopsis* (see Results). For a few genes that have undergone chromosomal translocation within *Drosophila*, the basal state for the genus, as reconstructed by Vibranovski et al. [31], was used. Chromosomal locations in the mosquito, *Anopheles gambiae*, were used to polarize gene movement on the basal *Teleopsis* branch that forms a split with *D. melanogaster*. We assumed a syntenic relationship between chromosome 3R in *A. gambiae* and the 2L/X chromosome in *D. melanogaster/Teleopsis* [70]. ‘Unknown’ character states for *Teleopsis* chromosomal location were assigned the state that minimized the amount of chromosomal gene movement and all genes that produced an ambiguous reconstruction were discarded. A measure of the rate of protein evolution for each gene was taken from the analysis of the *T. dalmanni* EST database [50] and was based on maximum likelihood trees constructed from amino acid data (using a JTT substitution model with no invariant sites) for *A. gambiae*, three *Drosophila* species—*D. melanogaster*, *D. pseudoobscura* and *D. virilis*—and *T. dalmanni*. The index measures the percentage of the entire tree length comprised by the branch leading to *T. dalmanni*, and was calculated by dividing the length of the branch leading to *T. dalmanni* by the length of the entire tree. We also used, from the Baker et al. [50] study, data on the number of putative gene duplications with the genus as well as data from a microarray experiment that compared the level of gene expression between males from two artificial lines selected for increased and decreased eye span. Over-representation of gene ontology terms was evaluated with GeneMerge [96], which uses a hypergeometric distribution and Bonferroni correction for multiple testing, to provide statistical rank scores for numerous functional categories. Statistical analyses were conducted with JMP [97].

### Linkage Mapping

Syntenic relationships for identified genes were established after constructing a linkage map based on anonymous microsatellites [98] and annotated EST loci [50] containing variable length tracts of repeated glutamines [99]. We conducted two F2 intercross experiments between different pairs of replicate lines of *T. dalmanni* selected for either long (high line) or short (low line) male relative eye span [cf. 40,99]. The first intercross was conducted after 32 generations of selection by mating a high line male with a low line female. This cross produced 490 flies, including 231 females and 259 males, in a single F2 family. For this analysis we genotyped 80 females and 95 males at 17 microsatellite and 26 amino acid repeat loci, of which 11 were X-linked and exhibited recombination. The second intercross was conducted after 45 generations of selection by mating a low line male with a high line female. The male used in this cross carried a drive X chromosome, which exhibits very little recombination with a standard X chromosome [40]. We used a single F2 family containing 464 flies including 271 females and 193 males. These flies were genotyped at 20 microsatellite and 18 amino acid repeat loci, of which four were X-linked.

For each family we used Joinmap v. 4.0 [100] to assign microsatellite and amino acid repeat loci to linkage groups using Kosambi map distances. We used the CP population type for autosomal loci and X-linked loci in females because this option allows for heterogeneous segregation types, resulting from a mixture of homozygous and heterozygous parents, and coded each locus to fit segregation expectations for the corresponding parental genotypes. We used haploid segregation expectations for X-linked loci in males. Because some microsatellite or annotated loci were informative in only one of the crosses, we used Joinmap to produce a joint linkage map, which combines the linkage maps for males and females in both families. A total of 17 microsatellite and 6 annotated loci were genotyped for all flies in both families and provide a common framework for estimating linkage relationships.

## Supporting Information

Table S1 Gene CGH log ratio values for the four *Teleopsis* species. In the majority of cases, gene names and IDs correspond to the annotation used for the *Drosophila melanogaster* genes that are homologous to the *Teleopsis* ESTs that were the source of the CGH probes. A few genes, that had no significant hit to *D. melanogaster*, use the gene names for their homolog in *Anopheles gambiae*, while those with no significant hit to any other gene, are designated with ‘ORF-’. *Td-T. dalmanni*, *Tw-T. white*, *Tt-T. thaii* and *Tq-T. quinqueguttata*. The inferred chromosomal locations (A-autosomal, X-X chromosome, and U-unknown) are presented for each gene. Log ratio values averaged across all four hybridization, as well as the individual log ratios for each hybridization, are also presented for each gene.
(1.76 MB XLS)

Table S2 Summary information on genotyped loci in *T. dalmanni*. Sample sizes and heterozygosity (Ho) for each sex, average CGH values, and linkage mapping in relation to chromosomal arm location in *D. melanogaster* (Dm) are provided. ‘Inf chr.’ provides the chromosomal location based on genotyping, while ‘CGH chr.’ provides the chromosomal location based on CGH. Linkage map value indicates chromosome and distance in cM (cf. Johns et al. [40]). These genes exhibit variation in amino acid repeats and were genotyped in outbred *T. dalmanni* flies as length variants by PCR.
(0.12 MB DOC)

Table S3 Summary of gene movement for *Teleopsis* species. Gene designations are explained in Table S1 legend. ‘Duplicate set’ refers to whether a duplication event was inferred for that gene within stalk-eyed flies based on an analysis of EST consensus sequences. For each gene, the inferred chromosomal locations (A-autosomal, X-X chromosome, and {AX}-unknown) for each *Teleopsis* species as well as their chromosomal location in *D. melanogaster* and *A. gambiae* are presented. The ‘Move’ column summarizes to which chromosome and on which branch of the tree the gene movement occurred. See Fig. 5 for identification of branch numbers and changes occurring at the base of the *Teleopsis* tree are designated ‘inTeleopsis’. Log ratio values averaged across all four hybridization for each *Teleopsis* species are also presented.
(0.07 MB XLS)
