# Supplementary material for: Comparative Genomic Hybridization (CGH) Reveals a Neo-X Chromosome and Biased Gene Movement in Stalk-Eyed Flies (Genus Teleopsis)
Source: PLoS Genet. 2010 Sep 16;6(9):e1001121. doi: 10.1371/journal.pgen.1001121 (PMC2940734; doi:10.1371/journal.pgen.1001121)
Supplement: Table S2 — Summary information on genotyped loci in T. dalmanni. Sample sizes and heterozygosity (Ho) for each sex, average CGH values, and linkage mapping in relation to chromosomal arm location in D. melanogaster (Dm) are provided. ‘Inf chr.’ provides the chromosomal location based on genotyping, while ‘CGH chr.’ provides the chromosomal location based on CGH. Linkage map value indicates chromosome and distance in cM (cf. Johns et al. [40]). These genes exhibit variation in amino acid repeats and were genotyped in outbred T. dalmanni flies as length variants by PCR. (0.12 MB DOC) [file pgen.1001121.s002.doc]

| Gene name | Male N | Male Ho | Female N | Female Ho | Inf  chr. | Linkage map | CGH -  avg(log_2_(F/M)) | CGH  chr. | Dm arm |
| --- | --- | --- | --- | --- | --- | --- | --- | --- | --- |
| bifocal | 46 | 0.67 | 47 | 0.79 | A | 1.115 | -0.041 | A | X |
| CG33691 | 84 | 0.57 | 82 | 0.62 | A | 1.115 | 0.008 | A | X |
| Tenascin major | 46 | 0.30 | 46 | 0.26 | A | 1.118 | 0.065 | A | 3L |
| BarH1 | 92 | 0.58 | 94 | 0.55 | A | 1.147 | 0.025 | A | X |
| grainy head | 43 | 0.40 | 45 | 0.40 | A | 1.247 | 0.002 | A | 2R |
| trachaeless | 93 | 0.31 | 88 | 0.38 | A | 1.381 | - | - | 3L |
| CG12104 | 45 | 0.27 | 46 | 0.35 | A | 1.589 | -0.013 | A | 3L |
| armadillo | 41 | 0.73 | 42 | 0.64 | A |  | -0.078 | A | X |
| Ecdysone-induced protein 75B | 46 | 0.11 | 46 | 0.13 | A |  | 0.038 | A | 3L |
| E5 | 43 | 0.44 | 43 | 0.49 | A | 2 | - | - | 3R |
| La related protein | 42 | 0.62 | 43 | 0.47 | A | 2.063 | 0.026 | A | 3R |
| corto | 81 | 0.72 | 74 | 0.64 | A | 2.08 | 0.138 | A | 3R |
| ptip | 45 | 0.47 | 45 | 0.53 | A | 2.106 | 0.076 | A | 3L |
| cap-n-collar | 45 | 0.47 | 46 | 0.50 | A | 2.135 | -0.070 | A | 3R |
| CG31224 | 36 | 0.19 | 38 | 0.24 | A | 2.221 | -0.123 | A | 3R |
| Band 4.1 inhibitor LRP interactor | 84 | 0.63 | 79 | 0.54 | A | 2.225 | -0.023 | A | 3R |
| CG4409 | 47 | 0.30 | 47 | 0.40 | A | 2.228 | -0.022 | A | 2R |
| domino | 92 | 0.77 | 87 | 0.71 | A | 2.236 | -0.021 | A | 2R |
| headcase | 47 | 0.43 | 42 | 0.33 | A | 2.248 | -0.043 | A | 3R |
| M-spondin (Q)* | 47 | 0.11 | 42 | 0.19 | A | 2.277 | 0.022 | A | 2R |
| mastermind | 45 | 0.22 | 45 | 0.27 | A | 2.287 | 0.142 | A | 2R |
| CG10321 | 44 | 0.61 | 46 | 0.65 | A | 2.352 | 0.051 | A | 2R |
| CG31064 | 84 | 0.52 | 81 | 0.53 | A | 2.412 | 0.017 | A | 3R |
| CG34347 | 44 | 0.50 | 47 | 0.70 | A | 2.46 | 0.111 | A | 3R |
| toutatis | 46 | 0.41 | 45 | 0.51 | A | 2.46 | 0.088 | A | 2R |
| CG10082 | 86 | 0.59 | 87 | 0.51 | A | 2.7 | 0.079 | A | 2R |
| CG10435 | 45 | 0.13 | 46 | 0.15 | A |  | -0.048 | A | 3R |
| Darkener of apricot | 46 | 0.52 | 46 | 0.43 | A |  | -0.037 | A | 3R |
| M-spondin (S)* | 46 | 0.26 | 44 | 0.20 | A |  | 0.022 | A | 2R |
| SRPK | 130 | 0.37 | 124 | 0.39 | A |  | 0.050 | A | 2R |
| bunched | 92 | 0 | 91 | 0.73 | X | 3.013 | 0.630 | X | 2L |
| CG8668 | 82 | 0 | 77 | 0.68 | X | 3.064 | 0.918 | X | 2L |
| CG42389 | 84 | 0 | 81 | 0.56 | X | 3.212 | 0.603 | X | 2L |
| cryptocephal | 131 | 0 | 127 | 0.62 | X | 3.561 | 0.891 | X | 2L |
| 3531953:1 | 83 | 0 | 80 | 0.64 | X |  | - | - | - |
| ORF-126 | 47 | 0 | 47 | - | Y | - | -6.732 | Y | - |

*- generated from different, non-overlapping consensus sequences that both have homology to M-spondin. The letter in parenthesis indicates the type of variable amino acid repeat.
